# Telemedicine in Elderly Hypertensive and Patients with Chronic Diseases during the COVID-19 Pandemic: A Systematic Review and Meta-Analysis

**DOI:** 10.3390/jcm12196160

**Published:** 2023-09-24

**Authors:** Miguel Quesada-Caballero, Ana Carmona-García, Sara Chami-Peña, Antonio M. Caballero-Mateos, Oscar Fernández-Martín, Guillermo A. Cañadas-De la Fuente, José Luis Romero-Bejar

**Affiliations:** 1 Centro de Salud Albayda La Cruz, Distrito Sanitario Granada-Metropolitano, Servicio Andaluz de Salud, Calle Virgen de la Consolación 12, 18015 Granada, Spain; miguel.quesada.caballero.sspa@juntadeandalucia.es; 2 Critical Care and Emergency Unit (UCCU), Distrito Sanitario Granada-Metropolitano, Servicio Andaluz de Salud, Calle Virgen de la Consolación 12, 18015 Granada, Spain; 3 Hospital de la Serranía de Ronda, Servicio Andaluz de Salud, Carretera San Pedro Km 2, 29400 Ronda, Spain; 4 Gastroenterology and Hepatology Department, San Cecilio University Hospital, Av. del Conocimiento s/n, 18016 Granada, Spain; 5 Centro de Salud Guadix, Área de Gestión Sanitaria Nordeste Granada, Servicio Andaluz de Salud, Ctra. de Murcia s/n, 18800 Baza, Spain; 6 Faculty of Health Sciences, University of Granada, Avda. Ilustración 60, 18016 Granada, Spain; gacf@ugr.es; 7 Brain, Mind and Behaviour Research Center (CIMCYC), University of Granada, 18071 Granada, Spain; 8 Statistics and Operational Research Department, University of Granada, Avda. Fuentenueva s/n, 18071 Granada, Spain; jlrbejar@ugr.es; 9 Institute of Mathematics, University of Granada (IMAG), Ventanilla 11, 18001 Granada, Spain; 10 Instituto de Investigación Biosanitaria (ibs.GRANADA), 18012 Granada, Spain

**Keywords:** chronic diseases, hypertension, older, telehealth, telemedicine

## Abstract

Background: One aspect of the distancing measures imposed in response to the COVID-19 pandemic is that telemedicine consultations have increased exponentially. Among these consultations, the assessment and follow-up of patients with chronic diseases in a non-presential setting has been strengthened considerably. Nevertheless, some controversy remains about the most suitable means of patient follow-up. Objective: To analyze the impact of the telemedicine measures implemented during the COVID-19 period on chronic patients. Material and Methods: A systematic review was carried out using the following databases: PubMed, Pro-Quest, and Scopus. The systematic review followed the guidelines outlined in the Preferred Reporting Items for Systematic Reviews and Meta-Analyses (PRISMA). The search equation utilized descriptors sourced from the Medical Subject Headings (MeSH) thesaurus. The search equation was: “hypertension AND older AND primary care AND (COVID-19 OR coronavirus)” and its Spanish equivalent. Results: The following data were obtained: 14 articles provided data on 6,109,628 patients and another 4 articles focused on a study population of 9684 physicians. Telemedicine was less likely to be used by elderly patients (OR 0.85; 95% C.I. 0.83–0.88; *p* = 0.05), those of Asian race (OR 0.69; 95% C.I. 0.66–0.73; *p* = 0.05), and those whose native language was not English (OR 0.89; 95% C.I. 0.78–0.9; *p* = 0.05). In primary care, lower use of telemedicine was associated with residents of rural areas (OR 0.81; *p* = 0.05), patients of African American race (OR 0.65, *p* = 0.05), and others (OR 0.64; *p* = 0.05). A high proportion (40%) of physicians had no prior training in telemedicine techniques. The highest quality in terms of telephone consultation was significantly associated with physicians who did not increase their prescription of antibiotherapy during the pandemic (OR = 0.30, *p* = 0.05) or prescribe more tests (OR 0.06 *p* = 0.05), i.e., who maintained their former clinical criteria despite COVID-19. Conclusions: Telemedicine is of proven value and has been especially useful in the COVID-19 pandemic. A mixed remote–presential model is most efficient. Appropriate training in this area for physicians and patients, together with correct provision, is essential to prevent errors in implementation and use.

## 1. Introduction

COVID-19 appeared in late 2019 in Wuhan (China), produced by a newly discovered coronavirus called SARS-CoV-2, and leading to the declaration of a health alert and pandemic in the first quarter of 2020 [1]. From the outset, social distancing was among the main measures employed to prevent infection [2,3]. This scenario has led to the development of non-presential consultations for the resolution of health problems while minimizing exposure to the virus, thus generating a boom in telemedicine [4].

Telemedicine had been employed before the appearance of the SARS-CoV-2, as an efficient form of medical consultation. An example of this is the advanced triage service, or teleconsultation, that had been operational for many years in American hospitals such as Jefferson Health, Mount Sinai, Cleveland Clinic, and Providence [5], a development that has been facilitated by technical and technological advances in medical science [6]. The first non-presential consultation was performed in 1905 by William Einthoven, who performed tele-electrocardiography and telephonic cardiac auscultation from his laboratory and from Leyden Hospital [7]. The technique is now widely used for patients with diabetes [8], chronic disease, or even mental health problems. In rural and semi-urban areas, access to medical services may be limited; elsewhere, access to consultations can also be restricted in order to avoid the spread of infection. The generalized lockdowns imposed during the recent pandemic compounded the problem [9]. For example, patients with hypertension experienced severe negative consequences for the control of their disease during the first months of the pandemic due to the restrictions imposed on mobility [10].

For all these reasons, technological solutions are increasingly being used to monitor patients remotely, especially for chronic patients and as a means of triaging potential patients with coronaviruses or other diseases in order to minimize the spread of infection [11]. Hypertension, the most prevalent chronic disease, is present in one third of the European population and in over 60% of patients aged 80 years or more, and is a risk factor for severe COVID-19 [12]. Home blood pressure monitoring enables telematically assessable follow-up, as an example of the practical value of non-presential healthcare [13]. However, despite its advantages, especially in the pandemic circumstances described, there remain areas in which telemedicine has little or no implementation, due to insufficient training or knowledge among healthcare professionals and/or users. Interestingly, chronic patients with hypertension have been among the least affected by the pandemic compared to patients with other conditions [14].

In view of these considerations, the use of telemedicine is expected to increase significantly to help manage the acute and chronic diseases experienced by an aging population whose longer life expectancy and better survival from cardiovascular events are reflected in a greater health burden. The use of remote consultations has played a major role among the healthcare measures implemented during the COVID-19 pandemic. Thanks to the establishment of classification and rapid response protocols, access and care for patients have been facilitated, minimizing the occurrence of complications and protecting the chronically ill and those over 80 years of age [15]. In the specific case of patients with hypertension, remote blood pressure monitoring has been highly successful, enabling faster patient follow-up and maintaining pre-pandemic levels of care [16].

The main aim of the present study is to analyze the use of telemedicine on the diagnosis, medical monitoring, and treatment of chronic hypertensive patients during the COVID-19 pandemic.

## 2. Materials and Methods

### 2.1. Design and Search Strategy

**Figure 1:**
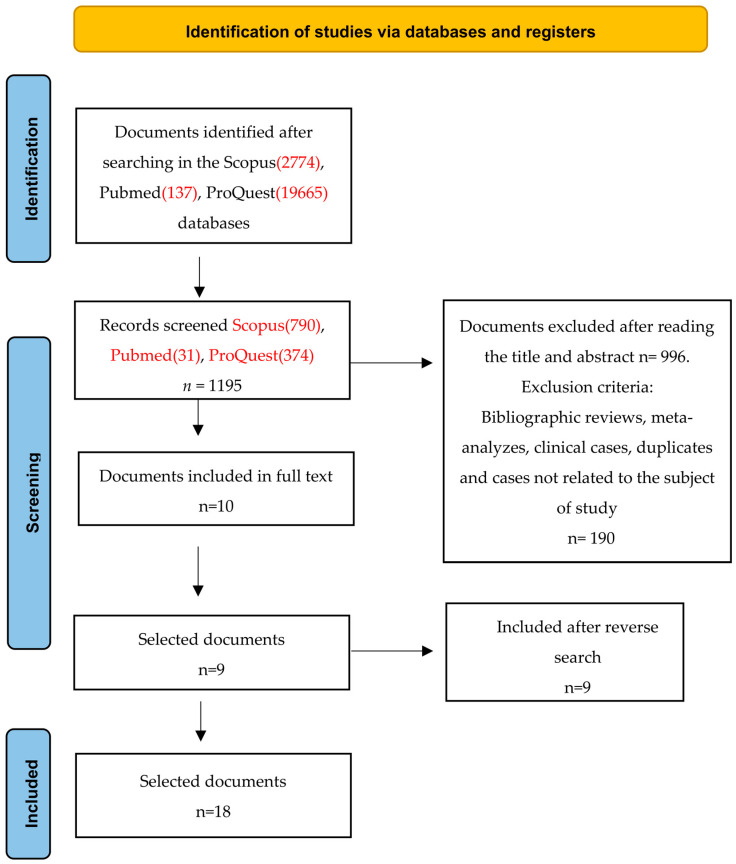
Flow diagram of the publication search process [17].

The review was conducted according to the Preferred Reporting Items for Systematic Reviews and Meta-Analyses (PRISMA) guidelines [17]. The following databases were consulted: PubMed, Pro-Quest, and Scopus. The Mesh terms employed in the search strategy were “hypertension AND older AND primary care AND (COVID-19 OR coronavirus)” and their equivalent in Spanish. The search was conducted in June 2023. Two reviewers participated in the screening process (see Figure 1). The study was registered (ID: 462362) in the PROSPERO database (International Prospective Register of Systematic Reviews).

### 2.2. Eligibility Criteria

Inclusion criteria: Primary quantitative studies investigating the impact of telemedicine on COVID respiratory syndrome pathology in hypertensive patients within a primary care setting, and published in either English or Spanish, were included without imposing any limitations on the publication year.

Exclusion criteria: Doctoral theses, articles without statistical information, duplicate studies, those not carried out in adults, or those whose main objective was not to investigate the relationship between telemedicine and its relationship with the consequences of the SARS-CoV-2.

### 2.3. Study Selection Process

Two team members (M.Q.-C. and A.M.C.-M.) conducted the search and study selection autonomously. If a discrepancy arose, a third researcher (A.C.-G.) was brought in for consultation. The article selection process involved four stages: (1) reviewing the title and abstract, (2) excluding articles not meeting the inclusion criteria, (3) examining the complete text, and (4) conducting a reverse search.

### 2.4. Data Extraction and Data Analysis

For data extraction from each study, a dedicated data collection notebook was crafted. This notebook encompassed essential information such as the primary author, publication year, study country, research design, sample details, the intervention upon which the study was centered, mean and standard deviation (SD), main results, and level of evidence. A descriptive analysis was done for the systematic review and two prevalence meta-analyses were performed, one about the satisfaction with the service of telemedicine and one about the prevalence of hypertension. The heterogeneity was assessed with I^2^ and publication bias with Egger test. The analysis was performed with the software StatsDirect.

### 2.5. Risk of Bias Assessment and Level of Evidence

The assessment of bias risk utilized the elimination questions derived from the CASP (Critical Appraisal Skills Program) specifically designed for cohort studies (Supplementary Table S1), including the studies with positive response to the three questions. In addition, we carried out the Egger test to assess the risk of bias. The evaluation of the quality of the studies incorporated into this review adhered to the levels of evidence and recommendation grades outlined by the Oxford Center for Evidence-Based Medicine (OCEBM) [18].

## 3. Results

### 3.1. Characteristic of the Studies Included

The initial search obtained 22,576 studies (PubMed 137; ProQuest 19,665; Scopus 2774), of which 323 were duplicates and discarded. After reading the title and abstract of each paper, 1195 (PubMed 31; ProQuest 374; Scopus 790) articles were selected for further analysis. Of these, 996 were not quantitative studies and a further 189 were unrelated to the subject of our investigation; all were excluded. Of the remaining 10 articles, 1 was excluded because it did not have access to full text, leaving 9. The reverse search procedure of the references cited obtained a further 9 eligible articles, resulting in a final total of 18 papers included in the definitive analysis [19,20,21,22,23,24,25,26,27,28,29,30,31,32,33,34,35,36] (see Figure 1).

These studies were carried out in the USA (six studies), Israel (three studies), Canada (two studies), and in Portugal, Norway, United Kingdom (UK), Chile, Saudi Arabia, Republic of Korea, and Spain (one study in each). Eight were cohort studies and eleven were transversal studies. Information on the characteristics of each study is shown in Table 1.

In total, 6,109,628 patients were included in these studies, but 5,791,812 corresponded to a single study [18]. In four papers [19,25,28,29,35], the study population was composed of doctors (total 9684, of which 7742 corresponded to a single study [28]).

In every case, hypertensive patients were included in the study samples analyzed, and in one paper they were exclusively analyzed as representative of patients with chronic disease [19].

### 3.2. Telemedicine, Consult Perception

According to the articles that considered patients’ opinions, most were highly satisfied with the telemedicine process. Thus, Gomes Almeida et al. (2020) found that 70.6% preferred this approach; Rodriguez-Fortúnez et al. [23] reported that 73.5% believed it optimized the management of their disease; and Zanaboni et al. [27] informed that for 72% of patients, telemedicine enhanced follow-up and for 58%, it improved the quality of treatment.

Eberly et al. [21] generated a patient profile according to which telemedicine was less commonly used by older patients (OR = 0.85; 95% C.I. 0.83–0.88; *p* = 0.05), those of Asian race (OR = 0.69; 95% C.I. 0.66–0.73; *p* = 0.05, those whose native language was not English (OR 0.89; 95% C.I. 0.78–0.9; *p* = 0.05), and those whose health insurance was provided by Medicaid (OR 0.93; C.I. 0.89–0.97; *p* = 0.05).

Ufholz et al. [33] reported that older patients showed less use of telemedicine (23%) despite near to 80% having access to technology.

Barayev et al. [25] observed that, for 60.7% of primary care physicians, WhatsApp^®^ consultations reduced the need for in-person visits at least once a week.

Chang et al. [26] observed more barriers to the use of telemedicine consultation in patients with a high Social Vulnerability Index (*p* = 0.001). Similarly, Dopelt et al. [24] established an association between literacy and patient satisfaction with the use of telemedicine (rp = 0.39 *p* = 0.001).

### 3.3. Telemedicine in COVID-19 Context

Gomes-de Almeida et al. [22] reported a reduction of 94.1% in face-to-face consultations by patients with hypertension and of 50.1% by patients with diabetes, while the use of telemedicine rose by 61.9%. Eberly et al. [21] focused on 148,402 appointment requests for telemedicine, of which 80,780 appointments actually took place; in 78,539 of these cases, the visit modality was specified.

Summers et al. [20] observed that many patients expressed concern about who might have access to their personal health data and about its future use (R = 0.916; *p* = 0.01). In addition, a strong correlation was recorded between people who wanted stronger data protection legislation and those who were worried about the re-use of the health data collected (R = 0.636; *p* = 0.01). Kaufman-Shriqui et al. [29] indicated that the majority of doctors who participated in their study (59.7%) carried out mixed (presential and telephone) counselling.

Lee et al. [19] observed a statistically significant decrease (*p* = 0.0001) in the continuity of care measured per physician and also in the number of physician visit days after the outbreak of COVID-19. The latter value decreased by 0.293 days, which, excluding the effects of telemedicine, amounts to 0.3330 days (*p* = 0.0001).

Dopelt et al. [24] addressed an 85% Internet-literate population, of whom 93% claimed to be well versed in e-health issues (in areas such as booking appointments and renewing prescriptions). However, only 38% of this population made use of the Internet for consultation or treatment sessions.

Dalbosco-salas et al. [36] showed that the telerehabilitation program implemented within primary healthcare has demonstrated feasibility and led to enhancements in physical capacity, quality of life, and symptom relief among adult COVID-19 survivors.

### 3.4. Telephone vs. Video Consultation

In a study of 78,539 telemedicine consultations, Eberly et al. [21] found that 54% were conducted by telephone and 46% by video call. The lower rate of video call use was associated with the following characteristics: patients of advanced age (OR = 0.79; *p* = 0.05), female sex (OR = 0.92; *p* = 0.05), African American race (OR = 0.65; *p* = 0.05), Hispanic race (OR 0.9; *p* = 0.05), and low socioeconomic status (OR = 0.57, *p* = 0.05 for income of <50,000 USD per year and OR 0.89, *p* = 0.05 for income of 50,000–100,000 USD per year).

Pierce and Stevermer [28] generated the following population profile for higher video call use: female sex (OR = 1.15, *p* = 0.05), age over 65 years (OR = 0.27, *p* = 0.05), African American race (OR = 0.72, *p* = 0.05), self-pay for healthcare (OR = 1.26, *p* = 0.05), or Medicaid (OR = 0.3,6 *p* = 0.05) or Medicare (OR = 0.79, *p* = 0.05). The authors also profiled a lower use of telemedicine consultation by patients living in rural areas (OR = 0.81, *p* = 0.05), those of African American race (OR = 0.65, *p* = 0.05), and those of other races (OR = 0.64, *p* = 0.05).

Chang et al. [26] reported that tele-calling was the main telehealth modality for the low Social Vulnerability Index (SVI) population, at 41.7% compared to 23.8% for the high SVI population (*p* = 0.01). Video calling, however, was the preferred modality in low SVI areas, at 33.7% (*p* = 0.01) compared to 18.7% in high SVI areas.

Juergens et al. [31] noted a total of 86,676 (41.5%) video consultations and 122,051 (58.5%) telephone consultations. Skin and soft tissue conditions exhibited the highest proportion of video visits (59.7%), while mental health conditions had the highest proportion of telephone visits (71.1%). Upon covariate adjustment, the overall rates of medication orders (46.6% vs. 44.5%), imaging orders (17.3% vs. 14.9%), lab orders (19.5% vs. 17.2%), and antibiotic orders (7.5% vs. 5.2%) were higher during video visits compared to telephone visits (*p* < 0.05). The most significant difference within the diagnostic groups was observed in skin and soft tissue conditions, where the rate of medication orders was 9.1% higher during video visits compared to telephone visits (45.5% vs. 36.5%, *p* < 0.05).

### 3.5. Doctors’ Use of Telemedicine vs. Presential Attention

Kaufman-Shriqui et al. [29], in a study focused on primary care, found that among physicians providing both in-person consultations and telemedicine during the COVID-19 pandemic, 40% had no prior training in the latter modality. They also reported that a higher quality of telephone consultation was significantly associated with doctors who did not increase their rates of antibiotherapy relative to the pre-pandemic situation (OR = 0.30, *p* = 0.05). Higher online quality was also associated with doctors who did not prescribe more tests than previously (OR = 0.06, *p* = 0.05).

Pierce and Stevermer [28] studied family doctors providing primary care in an out-of-hospital setting. These authors generated a user profile in which the lower use of telemedicine was associated with patients who were rural residents (OR 0.81, *p* = 0.05), of African American race (OR 0.65, *p* = 0.05), or of other races (OR = 0.64, *p* = 0.05).

Barayev et al. [25] analyzed 153 out-of-hospital family doctors and 48 in-hospital doctors, 86.9% and 86.5%, respectively, of whom used WhatsApp^®^ on a daily basis in a professional environment. Additional workload, possible breach of patient confidentiality, and lack of documentation on consultations were the main concerns among both groups of doctors. Nevertheless, 60.7% of primary care doctors and 95.7% of hospital specialists agreed that telemedicine is a useful tool for reducing the number of face-to-face appointments required. Finally, Chang et al. [26] studied the use of telemedicine in primary care in New York City in 2020 during the pandemic. These authors observed that telemedicine was provided unevenly and faced both patient- and provider-related barriers.

In a primary care investigation, Singer et al. [30] stated that there was no notable distinction in the quantity of follow-up visits administered in a clinic visit compared to a virtual care visit (8.7% vs. 5.8%) (*p* = 0.6496).

### 3.6. Meta-Analysis

**Figure 2:**
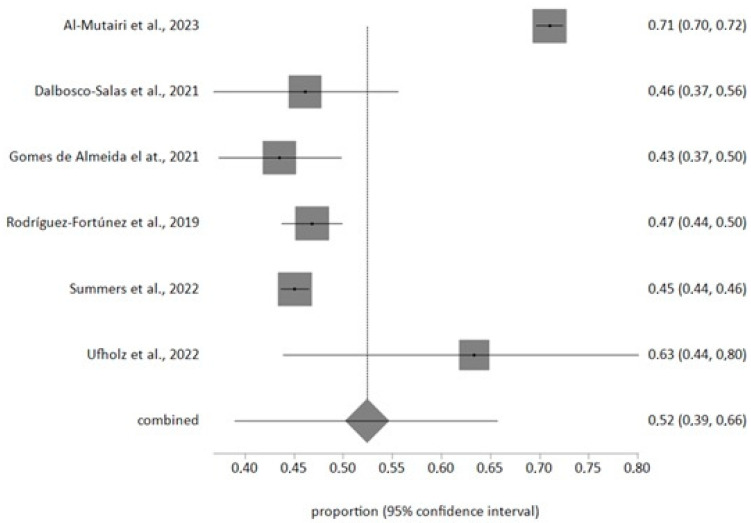
Forest plot of hypertension prevalence [20,22,23,32,33,36].

The meta-analysis of random effects about the prevalence of hypertension included 6 studies with a sample of n =10,464 and a meta-analytical estimation of 52%, CI 95% (39%, 66%) (Figure 2) and I^2^ value of 99.2%. The Egger test result was −3.97 (*p* > 0.5).

**Figure 3:**
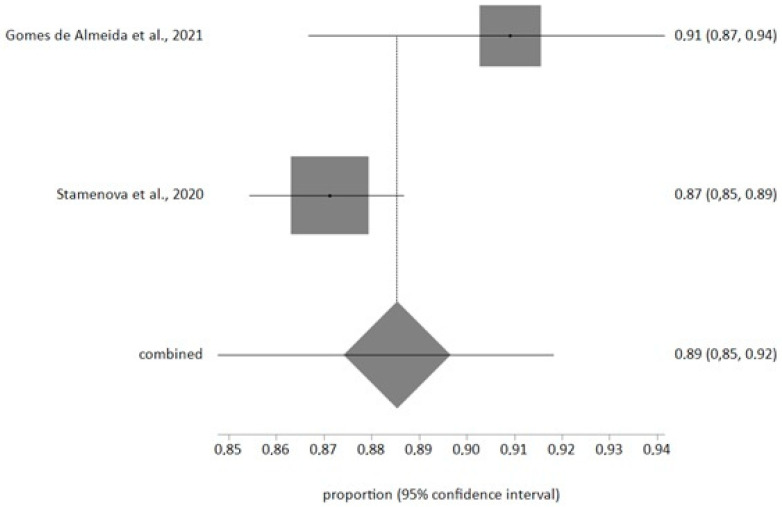
Forest plot of patients’ satisfaction with telemedicine prevalence [22,35].

The meta-analysis of random effects about the satisfaction with telemedicine included 2 studies with a sample of n = 1969 and a meta-analytical estimation of 89%, CI 95% (85%, 92%) (Figure 3), and I^2^ value of 69.3%. The Egger test result was −8.12 (*p* > 0.2). One included study analyzed the satisfaction with telemedicine with a Likert scale from 1 to 5 and the other analyzed satisfaction assessing the satisfaction comparing telemedicine quality with in-person visits.

## 4. Discussion

In this study, we analyze the role of telemedicine in its different formats. This form of healthcare had, to some extent, been employed previously, but became indispensable during the COVID-19 pandemic, as health systems worldwide struggled to meet the needs of users. Telemedicine was seen as the best way to minimize risks to healthcare staff and patients, and its use expanded exponentially [37,38].

In the last 50 years, medical care has undergone major changes, with the parallel provision of urgent and programmed care, together with novel processes to optimize treatments and follow-up. Primary healthcare, as the first link in the care chain, activates patient care and follow-up, while referral to specialists is the final step, whether or not an intervention finally takes place. In the latter case, depending on the severity of the illness, there must be subsequent re-evaluation and, if necessary, the continuation of treatment and/or prevention of recurrence. This is the case, for example, with acute or chronic processes such as cardiovascular problems, for which telematic monitoring, based on new technologies, provides a valuable treatment option [39,40,41].

The incorporation of a telematics approach can speed up healthcare processes by facilitating treatment continuity and follow-up in a non-presential manner, especially by means of telephone and video-call consultations [42]. The first approach is the most accessible, the cheapest, and the most widely used. It allows fast and efficient communication for simple consultations and is recommended for bureaucratic consultations such as prescription renewals, work incapacity reports, or requests for diagnostic tests for follow-up [43]. Video consultations have the advantage that the patient can be visualized, thus enabling a diagnosis to be more accurately approximated. This channel is objectively superior, and the equipment required is not complicated; nevertheless, the economic cost per consultation is higher and it is less widely used [44]. However, neither method is ideal for making a new diagnosis, as they do not allow the physician to conduct a complete exploration or to fully assess the patient’s non-verbal language, elements that can only be achieved in a face-to-face consultation [45].

The emergence of social networks and apps that expand the facilities available for human contact has given a new impetus to the use of remote communication, and their incorporation into telemedicine has facilitated the greater use of this approach. However, this must always be done with full regard for patient privacy and in compliance with data protection legislation, restrictions that can sometimes limit its use [46]. Currently, telemedicine is well accepted by patients, who find it as valid as in-person consultation, in part because the physician can attend more patients in a given period, and waiting times are reduced. Furthermore, telemedicine increases accessibility for patients with contagious diseases, as is the case with COVID-19 [47].

After the initial diagnosis, patients with hypertension can be monitored effectively by means of telemedicine [48]. However, although patient records and monitoring are acceptable with this approach, the quality of measurements obtained may decrease [49]. Smartphone apps, in conjunction with the self-monitoring of blood pressure, already form part of telemedicine, and have proven to be effective in providing discreet monitoring; their supervised use, in parallel with routine treatment, is recommended [50].

Finally, sufficient resources are currently available for the provision of telemedicine (mainly by telephone) throughout Europe, both in primary care and in hospitals. The studies reviewed are generally in accordance with this conclusion, although most have been carried out in western Europe, Canada, and the USA, while fewer data are available with respect to the use of telemedicine in eastern Europe. In fact, the trend worldwide and in the countries analyzed in this study demonstrates the growing interest in telehealth. Thanks to the study conducted during the pandemic in these countries, better health strategies and policies can be designed [51], which is much needed as previous studies show that telehealth infrastructures are inadequate [52]. The impact of the action protocols implemented in response to the COVID-19 pandemic has highlighted the remarkable value offered by the correct use of telemedicine [53]. On the other hand, in some medical specialties, its implementation has been less successful, with discrepancies between users’ expectations and the results obtained. One such case is the rehabilitation service, where treatment with inadequate equipment and/or materials can hinder rather than assist the patient’s recovery [54]. Therefore, the implementation of telemedicine must be carefully tailored to the needs of the healthcare system and to facilitate treatment, making sure that it is never the cause of under-diagnosis, misdiagnosis, or mistreatment. In other words, telemedicine should be applied to make the system more dynamic without losing the essence of the medical consultation.

The meta-analysis reported a prevalence estimation of 52%; this is in agreement with previous studies [55] but the American prevalence is high in older patient [56]. This situation may be due to there being new hypertensive limits in North America, 130/80 mmHg instead of 140/90 mmHg [57,58]; therefore, older patients do not have complete access to telemedicine and this is the population range with less telematic media use. Satisfaction has a very high level in our study and is similar to that in other similar articles [22,59]. The use of telemedicine improves the control and the contact with our patients and can be the reason for this high acceptance.

This study faced certain limitations. Initially, despite focusing on primary studies that analyzed the impact of telemedicine during the COVID-19 era, the diverse array of study designs and differing characteristics introduced heterogeneity in the outcomes. In addition, the fact that the studies were conducted in health systems in different countries may influence the variability of results. A meta-analysis was conducted; however, incorporating all the selected studies was unfeasible due to the substantial variability in parameters and analyzed variables.

Therefore, for future research, it is necessary to carry out more studies with larger samples and to analyze the evolution over time. It is also recommended that randomized clinical trials be conducted to explore in more depth how this care activity could benefit users.

## 5. Conclusions

The results imply that the utilization of telemedicine is an efficient means of diagnosis and follow-up, as has been demonstrated during the COVID-19 pandemic. Telephone consultation is the most widely used form of telemedicine, due to widespread telephone access and it being cheaper than other telematic medicine options.

However, appropriate provision and training for professionals and patients is needed in order to avoid problems in the implementation and application of resources. In-person consultations must continue to exist and will never be entirely replaced by telemedicine, which can be of great assistance, for example by facilitating follow-up, but also presents significant shortcomings that cannot be overcome.

Because of this, it is advisable to create more guidelines for telemedicine, improve knowledge, and improve telemedicine implementation, and future investigation will be necessary for a correct medical assistance.

## Figures and Tables

**Table 1:** Information on the selected studies.

Author, Year, Country	Type of Study	Sample	Intervention	Main Findings	Conclusions	EL/RG
Al-Mutairi et al., 2023 (Saudi Arabia) [32]	Retrospective, cohorts	4266 patients	Coronavirus disease 2019 (COVID-19) pandemic in Saudi Arabia (March 2020 to June 2020) shifted routine in-person care for patients with type 2 diabetes mellitus (DMT2) to telemedicine. The aim of this study was to investigate the impact telemedicine had during this period on glycemic control (HbA1c) in patients with DMT2 and with AHT and older as a comorbilities in almost 50% of patients.	The patient demographics consisted primarily of Saudis (97.7%), with 59.7% being female and 56.4% aged ≥60 years. The prevalence of obesity was 63.8%, dyslipidemia was 91%, and hypertension was 70%. The mean HbA1c for all patients showed a slight increase from 8.52% ± 1.5% before the lockdown to 8.68% ± 1.6% after the lockdown. Among the patients, n = 1064 (24.9%) witnessed a decrease in HbA1c by ≥0.5%, n = 1574 patients had an increase in HbA1c by ≥0.5% (36.9%), and n = 1628 patients experienced an HbA1c change of <0.5% in either direction (38.2%). Notably, a greater percentage of males demonstrated significant improvements in glycemia compared to females (28.1% vs. 22.8%, *p* < 0.0001), as did individuals below the age of 60 years (28.1% vs. 22.5%, *p* < 0.0001). Hypertensive individuals were less likely to experience glycemic improvement than non-hypertensive individuals (23.7% vs. 27.9%, *p* = 0.015). Patients on sulfonylureas exhibited a higher proportion of HbA1c improvement (42.3% vs. 37.9%, *p* = 0.032), while patients on insulin had higher HbA1c levels (62.7% vs. 56.2%, *p* = 0.001). The changes in HbA1c were independent of BMI, hyperlipidemia, disease duration, and cardiac or renal conditions.	Telemedicine proved effective in providing care to patients with type 2 diabetes during the COVID-19 lockdown; 63.1% of patients maintaining their HbA1c levels and achieving better glycemic control. Improvement was notably higher among males compared to females. Despite these advances, HbA1c levels remained persistently elevated in these patients before and after blockade. Although the improvement was greater among males than females, HbA1c levels remained elevated in these patients before and after blockade. This problem is probably due to factors related to healthy lifestyle, age, education, and hypertension.	2a/B
Barayev et al., 2021 (Israel) [25]	Cross-sectional	201 doctors	Cross-sectional study based on an anonymous web survey conducted among family doctors and hospital physicians working in the Israel Defence Forces Medical Corps, during September and October 2019.	A total of 153 participants were family physicians and 48 were hospital specialists. WhatsApp^®^ is used daily in professional settings by 86.9% of PCPs and by 86.5% of hospital specialists. The additional workload, potential breaches of patient confidentiality, and lack of complete documentation of consultations were the main concerns expressed about the app. However, 60.7% of PCPs and 95.7% of specialists stated that it enabled them to reduce in-person consultations at least once a week.	In the social distancing required by COVID-19, WhatsApp^®^ offers a simple and readily available platform for consultations between healthcare providers, making some in-person appointments unnecessary. However, some issues remain to be addressed, such as patient confidentiality, the possible lack of documentation of patients’ medical history, and the need to compensate those who provide telemedicine services after business hours.	2c/B
Chang et al., 2021 (USA) [26]	Cross-sectional	1100 doctors	Analysis of telemedicine use and barriers to use in small primary care practices. The information comes from surveys conducted by the New York City Department of Health and Mental Hygiene and New York University. The purpose of these surveys was to understand the strategies and responses of primary care practices in the midst of the COVID-19 pandemic. The collection was conducted between 10 April and 18 June 2020.	Healthcare practitioners in regions characterized by high Social Vulnerability Index (SVI) were nearly twice as likely to predominantly employ telephones as their primary telemedicine mode (41.7% vs. 23.8%; *p* < 0.001), compared to their counterparts in low SVI regions. Conversely, video-based telemedicine was predominantly utilized by 18.7% of providers in high SVI areas, contrasting with 33.7% in low SVI areas (*p* < 0.001). Moreover, healthcare providers in high SVI areas encountered more patient-related barriers but fewer obstacles on the provider’s end, as opposed to those in low SVI areas.	Telemedicine became an important mode of primary care delivery in New York City during the pandemic. Nevertheless, the shift towards telemedicine was not uniform across all communities. To promote more equitable access in this realm, policy adjustments should aim to tackle the impediments experienced by underserved populations, including both patients and caregivers.	2c/B
Dalbosco-Salas et al., 2021 (Chile) [36]	Cohorts	115 patients	The study assessed the efficacy of a telerehabilitation program implemented within primary care for post-COVID-19 patients. An observational, prospective study was carried out across seven primary care centers in Chile, encompassing adult patients (>18 years) with a history of SARS-CoV-2 infection.	The telerehabilitation program comprised 24 sessions of supervised exercise training conducted at patients’ homes. Its effectiveness was evaluated using the 1-min Sit-to-Stand Test (1-min STST), SF-36 questionnaire, fatigue levels, and dyspnea symptoms before and after the intervention. The study enrolled 115 patients, with 55.4% being female, and a mean age of 55.6 ± 12.7 years. Among them, 57 patients (50%) had a history of hospitalization, and 35 (30.4%) were ICU admissions. Following the intervention, the 1-min STST showed improvement, increasing from 20.5 ± 10.2 (53.1 ± 25.0% predicted) to 29.4 ± 11.9 (78.2 ± 28.0% predicted) repetitions (*p* < 0.001). Additionally, the SF-36 global score demonstrated a significant enhancement, rising from 39.6 ± 17.6 to 58.9 ± 20.5.	Fatigue and dyspnea exhibited significant improvement post-intervention. Despite the lack of a control group, this report demonstrated the viability and effectiveness of a tele-rehabilitation program implemented within primary healthcare. It notably enhanced physical capacity, quality of life, and symptom management in adult survivors of COVID-19.	2a/B
Dopelt et al., 2021 (Israel) [24]	Cross-sectional	156 doctors	This study examined the extent of telemedicine use and the relationship between eHealth literacy and satisfaction with telemedicine use during the pandemic. A total of 156 patients at a clinic in southern Israel completed an online questionnaire.	In the sample, 86% of participants could use the Internet to obtain health information, but only one-third felt confident in using it to make health decisions. Further, 93% used the Internet for technical actions, such as renewing prescriptions or making appointments. Only 38% used telemedicine for consultations or treatment sessions. eHealth literacy and satisfaction were positively associated with telemedicine use (rp = 0.39, *p* < 0.001). Although respondents understood the benefits of telemedicine, they were neither satisfied with nor interested in online sessions once the COVID-19 epidemic became less acute, preferring in-person meetings. Young people and academics benefit most from telemedicine, creating gaps in use and potentially increasing healthcare inequality.	Intervention programs should be developed, especially among vulnerable populations, to strengthen e-health literacy and remove barriers that may generate scepticism about the use of telemedicine, during and after the pandemic.	2c/B
Eberly et al., 2020 (USA) [21]	Retrospective, cohorts	148,402 patients with scheduled appointments; 80,780 appointments kept.	The association between video and telephone consultations with gender, race, language, and socioeconomic status was studied, from 16 March to 11 May 2020.	Of 78,539 consultations, 35,824 were by video and 42,715 by telephone. Lower use of telemedicine was associated with: 1-Advanced age OR 95% CI 0.85 (0.83–0.88). 2-Asian race OR 95% CI 0.69 (0.66–0.73). 3-Preferred language other than English OR 95% CI 0.89 (0.78–0.9). 4-Medicaid insured OR 95% CI 0.93 (0.89–0.97). Lower use of video visits was associated with 1-Advanced age OR 95% CI 0.79 (0.76–0.82) 2-Female sex OR 95% CI 0.92 (0.9–0.95) 3-Black race OR 95% CI 0.65 (0.62–0.68) 4-Hispanic Race OR 95% CI 0.9 (0.83–0.97) 5-Low socioeconomic level OR 95% CI 0.57 (0.54–0.60) (less than 50,000 USD) and OR 95% CI 0.89 (0.85–0.92) (50,000–100,000 USD).	During the COVID-19 pandemic, patients who were older, Asian, or non-English speaking made less use of telemedicine, while older patients, women, African Americans, Latinos, and poorer patients used video calls more. Access to telemedicine is unequal, which should be investigated further.	2a/B
Gomes-de Almeida et al., 2021 (Portugal) [22]	Cross-sectional	253 patients	Patient satisfaction was studied on 4–5 January 2020 using a questionnaire scored on a Likert scale.	In the sample, 70.6% of patients prefer telemedicine. Consultations for diabetes fell by 50.1% and for hypertension by 94.1%, compared with pre-COVID. Telemedicine consultations rose by 61.9%.	The vast majority of telemedicine users during the COVID-19 pandemic were satisfied with the results.	2c/B
Juergens et al., 2022 (USA) [31]	Cross-sectional	809,146 completed adult primary care telemedicine encounters.	In this study, patients who autonomously scheduled and successfully participated in telemedicine appointments with their designated primary care provider or an alternate available primary care provider within the same medical group were identified. The data collection encompassed the period from 1 April 2020 to 31 October 2020, during which physical distancing measures due to COVID-19 were enforced.	A total of 273,301 encounters were analyzed, comprising 86,676 (41.5%) video visits and 122,051 (58.5%) telephone visits. In terms of specific diagnosis groups, skin and soft tissue conditions exhibited the highest proportion of video visits (59.7%), whereas mental health conditions had the highest proportion of telephone visits (71.1%). Upon covariate adjustment, the overall rates of medication orders (46.6% vs. 44.5%), imaging orders (17.3% vs. 14.9%), lab orders (19.5% vs. 17.2%), and antibiotic orders (7.5% vs. 5.2%) were notably elevated during video visits in comparison to telephone visits (*p* < 0.05). The most significant difference within diagnosis groups was observed in skin and soft tissue conditions, where the rate of medication orders during video visits was 9.1% higher than during telephone visits (45.5% vs. 36.5%, *p* < 0.05).	The study showed notable and statistically significant variations in clinician orders based on the type of visit in telemedicine encounters, particularly for prevalent primary care conditions. The results strongly indicate that, for specific conditions, the visual information provided during video visits can facilitate clinical assessment and treatment decisions.	2c/B
Kaufman-Shriqui et al., 2022 (Israel) [29]	Cross-sectional	159 family doctors	The study was conducted using a 47-item online Google Crosswalk survey, via the Israel Association of Family Physicians mailing list, between 31 March and 5 May 2020. The questionnaire obtained demographic data, physician characteristics, and information on the use and perceived quality of telemedicine.	The use of telephone consultation by physicians was inversely associated with their prescribing antibiotics during the COVID-19 pandemic (OR 0.30 95% CI (0.134–0.688) *p* = 0.004) and with their requesting more blood tests during the pandemic (OR = 0.06 95% CI (0.008–0.378) *p* = 0.003).	Telemedicine has considerable promise in primary care and has great potential for improvement. However, the interpersonal challenges need to be thoroughly understood so that physicians can receive personalized coaching. Further randomized trials are needed, including patient-reported outcomes. Research is also needed on the utility, cost, and cost-effectiveness of telemedicine for follow-up, prescribing, and additional referrals.	2c/B
Khairat et al., 2020 (USA) [34]	Cohorts	733 virtual visits	The objective of this study was to investigate the patterns of confirmed COVID-19 cases in North Carolina and to comprehend the trends in virtual visits associated with symptoms of COVID-19.	By 18 March 2020, a total of 92 confirmed COVID-19 cases and 733 virtual visits were documented. Out of these virtual visits, 257 (35.1%) were linked to symptoms resembling COVID-19. Among these visits, the majority were by females (178 visits, 69.2%). Patients aged between 30 and 39 years (n = 67, 26.1%) and 40 and 49 years (n = 64, 24.9%) constituted half of the total cases. Remarkably, almost 96.9% (n = 249) of the COVID-like encounters were reported within the state of North Carolina. The study underscores the efficiency of virtual care in effective triage, especially in counties with a high incidence of COVID-19 cases. Furthermore, it affirms that the disease spreads extensively in densely populated regions and areas with major airports.	The utilization of virtual care holds significant promise in combating the COVID-19 pandemic. It has the potential to diminish emergency room visits, preserve critical healthcare resources, and mitigate the spread of COVID-19 by enabling remote patient treatment. The findings from this study strongly advocate for the widespread integration of virtual care within global health systems as a crucial approach in addressing the challenges posed by the ongoing COVID-19 pandemic.	2a/B
Lee et al., 2022 (Korea) [19]	Cohorts	5,791,812 hypertensive patients	The aim of this study was to assess the impact of COVID-19 on the continuity of care of hypertensive patients, considering the use of telemedicine. Study data were obtained from insured physicians in South Korea, for 2019 and 2020.	After the outbreak of COVID-19 and the increased use of telemedicine, in-person consultations decreased by 0.293 days (*p* < 0.0001) to 0.333 days per patient.	COVID-19 protocols did not affect treatment continuity for patients with hypertension, but did affect the frequency of outpatient visits. Medical care was not interrupted, but there was a significant difference in the type of medical care provided, with the inclusion of telemedicine.	2a/B
Pierce and Stevermer 2020 (USA) [28]	Cross-sectional	7742 family doctors	The aim of the study was to study family medicine visits at a single US institution in the initial month of the COVID-19 public health emergency (17 March to 16 April 2020), comparing the demographics of patients using telemedicine with those using in-person visits during the same period, and the demographics of those using full audio and video with those using audio only.	The likelihood of any telemedicine visit in the first 30 days of its expansion was higher for women (OR 1.15 95% CI 1.04–1.26), persons aged 65 years or older (OR 1.21 95% CI 1.05–1.40), self-pay patients (OR 1.26 95% CI 1.04–1.52), and those with Medicaid (OR 1.29 95% CI 1.04–1.61) or Medicare (OR 1.37 95% CI 1.18–1.60) as primary payers. The likelihood of a telemedicine visit was lower for rural residents (OR 0.81 95% CI 0.74–0.90), persons of African American race (OR 0.65 95% CI 0.56–0.75) or of other races (OR 0.64 95% CI 0.50–0.82). The likelihood of a complete telemedicine visit with audio and video was lower for patients who were older (OR 0.27 95% CI 0.21–0.33), of African American race (OR 0.72 95% CI 0.55–0.93), from urban areas (OR 1.36 95% CI 1.14–1.61), or self-pay, or with Medicaid (OR 0.36 95% CI 0.26–0.51) or Medicare (OR 0.79 95% CI 0.64–0.99).	Age, race, area of residence, and insurance provision are significant variables for the use of telemedicine in the context of the COVID-19 pandemic.	2c/B
Rodríguez-Fortúnez et al., 2019 (Spain) [23]	Cross-sectional	1036 patients	Observational, cross-sectional study conducted among diabetics over 18 years of age with data for one year, conducted between 18 April and 5 May 2016.	Blood glucose values were recorded by 85.9% of patients, but data for lifestyle habits by only 14.4%. Previous experience with telemedicine was reported by 9.8% of patients, of whom 70.5% were satisfied with the service while 73.5% considered that the use of telemedicine had optimized their DM2 management. However, most remarked on areas for improvement, such as ease of use (81.4%), interaction with the medical team (78.4%), and the time required for data recording/transfer (78.4%). Experienced patients had better perceptions of the usefulness of telemedicine than naïve patients, for all aspects considered (*p* < 0.05).	In Spain, almost 10% of patients with DM2 have experience with telemedicine, and it is well accepted, especially when based on glucometers. However, to expand the use of telemedicine, easier and time-saving programs for patient–physician interaction should be implemented.	2c/B
Singer et al., 2022 (Canada) [30]	Retrospective, cohorts	142,616 patients 154 primary care providers	This study analyzed the characteristics of virtual visits, providing insights into the utilization and users of virtual care within primary care settings during the COVID-19 pandemic, specifically from 14 March 2020, to 30 June 2020.	Between 14 March 2020 and 30 June 2020, a total of 146,372 visits were administered by 154 primary care providers. Among these, 33.6% were conducted via virtual care. Female patients (OR 1.16, CI 1.09–1.22), patients with ≥3 comorbidities (OR 1.71, CI 1.44–2.02), and patients with ≥ 10 prescriptions (OR 2.71, 2.2–1.53) had a higher likelihood of having at least one virtual care visit compared to male patients, those with no comorbidities, and those with no prescriptions. Notably, the study found no significant difference in the number of follow-up visits provided as a clinic visit compared to a virtual care visit (8.7% vs. 5.8%) (*p* = 0.6496).	In the early stages of the pandemic restrictions, about a third of visits were conducted virtually. Patients with a higher number of comorbidities and prescriptions were the ones predominantly utilizing virtual care, suggesting that patients with chronic disease requiring ongoing care utilized virtual care. Virtual care as a primary care visit type continues to evolve. Ongoing provision of virtual care can enhance quality, patient-centered care moving forward	2a/B
Stamenova et al., 2020 (Canada) [35]	Retrospective, cohorts	14,291 patients 326 primary care providers	Restrospective cohorts study with the aim to assess the adoption of a virtual visit platform in primary care, investigate the preferences of patients and physicians regarding virtual communication methods, and provide insights into visit characteristics and patient experiences with the care provided.	A total of 44% of registered patients and 60% of registered providers actively utilized the platform. Among the patients, 51% successfully completed at least one virtual visit. Interestingly, a vast majority of these virtual visits (94%) primarily utilized secure messaging. Notable patient requests included medication prescriptions (24%) and follow-up from a previous appointment (22%). Conversely, providers frequently used virtual visits to follow up on test results (59%). Impressively, 81% of virtual visits, according to providers, required no further follow-up for the respective issue. Moreover, an overwhelmingly positive response was received, with 99% of patients expressing their intent to use virtual care services again.	Although the availability of primary care video visit services is on the rise, this study revealed a noteworthy preference for secure messaging over video among both patients and providers in rostered practices. Contrary to concerns about potential overuse of virtual visits, it was observed that virtual visits, especially when patients connected with their designated primary care provider, often substituted in-person visits without overwhelming physicians with excessive requests. This approach shows promise in enhancing access and maintaining continuity in primary care services.	2a/B
Summers et al., 2022 (UK) [20]	Cohorts	4764 patients	Study conducted by email invitation (11,213 sent) of patients over 18 years of age and registered at Diabetes.co.uk. Data were collected 6–31 August 2020, including quantitative information on demographic characteristics, COVID-19 diagnosis and symptoms, privacy and custody of pre- and post-COVID-19 health data, and COVID-19 blocking behavior. The study aim was to determine patients’ willingness to share health data during the COVID-19 pandemic.	N(1) DMT2 = 2974 = 62.7%. N(2) AHT = 2147 = 45.2% N(3) DMT1 = 1299 = 27.4%. A positive correlation was observed between concern about the future use of clinical data and concern about data access (R = 0.916; *p* = 0.01). There was a strong correlation between concern about the need for stronger legislation and concern about the reuse of shared health data (R = 0.636 *p* = 0.01).	Data sensitivity is highly contextual. Most participants are more comfortable sharing anonymized rather than personally identifiable data. Willingness to share data also depends on the receiving agency; trust is a key issue, in particular, concerning who may access shared personal health data and how it may be used.	2a/B
Ufholz et al., 2023 (USA) [33].	Cross-sectional	30 patients	The objective of this study was to conduct a survey among elderly primary care patients, focusing on their readiness for telemedicine. This included assessing their familiarity with Internet usage, possession of Internet-capable devices, past experiences, and concerns regarding telemedicine, as well as identifying perceived barriers. The findings from this survey were utilized to develop a telemedicine preparedness training program. The patients were 65–81 years old.	The majority of participants (21 out of 30, 70%) stated that they possessed a device suitable for telemedicine and utilized the Internet. However, approximately half of them had only one connected device, with messaging and video calling being the applications most frequently utilized. Email usage was minimal, and no one used online shopping or banking services. Merely 7 patients had engaged in telemedicine appointments. Telemedicine users tended to be younger than nonusers and utilized a greater range of Internet functions. Only 2 individuals reported encountering issues during their telemedicine sessions (related to technology and privacy). Almost all respondents acknowledged the advantages of telemedicine in terms of avoiding travel and reducing exposure to COVID-19. The most prevalent concerns were the potential loss of the doctor–patient connection and the inability to undergo a physical examination.	The majority of elderly individuals mentioned owning devices suitable for telemedicine; however, their Internet usage patterns did not validate the sufficiency of their devices or skills for telemedicine. While doctor–patient discussions could assist in addressing telemedicine apprehensions, it is imperative to also tackle gaps in device functionality and digital proficiency.	2c/B.
Zanaboni and Fagerlund, 2020 (Norway) [27]	Cross-sectional	2043 patients	An online survey obtained quantitative and qualitative data to determine: (1) user characteristics; (2) use; (3) experiences, perceived benefits and satisfaction; and (4) time spent using telemedicine services. Conducted from January 2017 to April 2018.	Women, young adults, and digitally active citizens with higher education were most likely to use telemedicine. A significant 80% of individuals found it simpler and more efficient to schedule appointments electronically compared to using the phone. An impressive 90% of the surveyed participants believed that renewing a prescription electronically was a hassle-free process. Additionally, 76% stated that managing their medications electronically provided them with a clearer understanding, and a notable 46% reported higher adherence to their prescribed medication regimen. For non-clinical visits, 60% of respondents found emails easier than communicating by phone. For clinical consultations, 72% agreed that electronic consultation improved follow-up, and 58% associated it with better treatment. These users were very satisfied with telemedicine services and would recommend them. The main benefit cited was time saving, which was confirmed by an objective comparison of time spent using telemedicine services vs. conventional approaches.	In Norway, users of e-consultation with family doctors and other digital health services are generally satisfied and consider these tools effective and efficient options to the usual forms of consultation.	2c/ B

AHT: arterial hypertension; CI: confidence interval; DMT1: diabetes mellitus type 1; DMT2: diabetes mellitus type 2; EL: evidence level; HbA1c: glycosylated hemoglobin; ICU: intensive care unit; OR: odds ratio; PCPs: primary care physicians; RG: research grade; SF-36: 36 Item Short Form Health Survey; SVI: Social Vulnerability Index; STST: sit-to-stand test; UK: United Kingdom; USA: United States of America.

## Supplementary Materials

The following supporting information can be downloaded at: https://www.mdpi.com/article/10.3390/jcm12196160/s1, Table S1: Critical reading of included studies.

## References

[1] WHO Guidance on Routine Immunization Services during COVID-19 Pandemic in the WHO European Region, 20 March 2020 n.d. https://www.who.int/europe/publications/i/item/WHO-EURO-2020-1059-40805-55114.

[2] CDC How to Protect Yourself and Others|CDC n.d. https://www.cdc.gov/coronavirus/2019-ncov/prevent-getting-sick/prevention.html?CDC_AA_refVal=https%3A%2F%2Fwww.cdc.gov%2Fcoronavirus%2F2019-ncov%2Fprevent-getting-sick%2Fsocial-distancing.html#stay6ft.

[3] Guillem F.C. (2020). Opportunities and threats for prevention and health promotion and the PAPPS in the context of the COVID-19 pandemic. Aten. Prim..

[4] Magoon V. (2020). Operationalizing virtual visits during a public health emergency. Fam. Pract. Manag..

[5] Hollander J.E., Carr B.G. (2020). Virtually Perfect? Telemedicine for COVID-19. N. Engl. J. Med..

[6] Jagarapu J., Savani R.C. (2021). A brief history of telemedicine and the evolution of teleneonatology. Semin. Perinatol..

[7] Hjelm N.M., Julius H.W. (2005). Centenary of tele-electrocardiography and telephonocardiography. J. Telemed. Telecare.

[8] Horton M.B., Brady C.J., Cavallerano J., Abramoff M., Barker G., Chiang M.F., Crockett C.H., Garg S., Karth P., Liu Y. (2020). Practice Guidelines for Ocular Telehealth-Diabetic Retinopathy, Third Edition—PubMed (nih.gov). Telemed. J. e-Health.

[9] Cullington H., Kitterick P., Darnton P., Finch T., Greenwell K., Riggs C., Weal M., Walker D.M., Sibley A. (2022). Telemedicine for Adults with Cochlear Implants in the United Kingdom (CHOICE): Protocol for a Prospective Interventional Multisite Study. JMIR Res. Protoc..

[10] Gotanda H., Liyanage-Don N., Moran A.E., Krousel-Wood M., Green J.B., Zhang Y., Nuckols T.K. (2022). Changes in blood pressure outcomes among hypertensive individuals during the COVID-19 pandemic: A time series analysis in three US healthcare organizations. Hypertension.

[11] Mandrioli J., Santangelo M., Luciani A., Toscani S., Zucchi E., Giovannini G., Martinelli I., Cecoli S., Bigliardi G., Scanavini S. (2021). TeleNeurological evaluation and Support for the Emergency Department (TeleNS-ED): Protocol for an open-label clinical trial. BMJ Open.

[12] Underlying Medical Conditions Associated with Higher Risk for Severe COVID-19, Information for Healthcare Professionals|CDC n.d. https://www.cdc.gov/coronavirus/2019-ncov/hcp/clinical-care/underlyingconditions.html.

[13] Reuter H., Jordan J. (2019). Status of hypertension in Europe. Curr. Opin. Cardiol..

[14] Varandani S., Nagib N.D. (2022). Evaluating the impact of the COVID-19 pandemic on monthly trends in primary care. Cureus.

[15] Omboni S., McManus R.J., Bosworth H.B., Chappell L.C., Green B.B., Kario K., Logan A.G., Magid D.J., Mckinstry B., Margolis K.L. (2020). Evidence and recommendations on the use of telemedicine for the management of arterial hypertension: An international expert position paper. Hypertension.

[16] Armitage L.C., Lawson B.K., Roman C., Thompson B., Biggs C., Rutter H., Lewis-Jones M., Ede J., Tarassenko L., Farmer A. (2022). Ambulatory blood pressure monitoring using telemedicine: Proof-of-concept cohort and failure modes and effects analyses. Wellcome Open Res..

[17] Page M.J., McKenzie J.E., Bossuyt P.M., Boutron I., Hoffmann T.C., Mulrow C.D., Shamseer L., Tetzlaff J.M., Akl E.A., Brennan S.E. (2021). The PRISMA 2020 statement: An updated guideline for reporting systematic reviews. BMJ.

[18] Howick J., Chalmers I., Glasziou P., Greenhalg T., Heneghan C., Liberati A., Moschetti I., Phillips B., Thornton H. (2011). The Oxford Levels of Evidence. https://www.cebm.net/2016/05/ocebmlevels-of-evidence.

[19] Lee S.Y., Chun S.Y., Park H. (2022). The impact of COVID-19 protocols on the continuity of care for patients with hypertension. Int. J. Environ. Res. Public Health.

[20] Summers C., Griffiths F., Cave J., Panesar A. (2022). Understanding the security and privacy concerns about the use of identifiable health data in the context of the COVID-19 pandemic: Survey study of public attitudes toward COVID-19 and data-sharing. JMIR Form. Res..

[21] Eberly L.A., Kallan M.J., Julien H.M., Haynes N., Khatana S.A.M., Nathan A.S., Snider C., Chokshi N.P., Eneanya N.D., Takvorian S.U. (2020). Patient characteristics associated with telemedicine access for primary and specialty ambulatory care during the COVID-19 pandemic. JAMA Netw. Open.

[22] Gomes-de Almeida S., Marabujo T., do Carmo-Gonçalves M. (2021). Telemedicine satisfaction of primary care patients during COVID-19 pandemics. Semergen.

[23] Rodríguez-Fortúnez P., Franch-Nadal J., Fornos-Pérez J.A., Martínez-Martínez F., de Paz H.D., Orera-Peña M.L. (2019). Cross-sectional study about the use of telemedicine for type 2 diabetes mellitus management in Spain: Patient’s perspective. The EnREDa2 Study. BMJ Open.

[24] Dopelt K., Avni N., Haimov-Sadikov Y., Golan I., Davidovitch N. (2021). Telemedicine and eHealth literacy in the era of COVID-19: A cross-sectional study in a peripheral clinic in Israel. Int. J. Environ. Res. Public Health.

[25] Barayev E., Shental O., Yaari D., Zloczower E., Shemesh I., Shapiro M., Glassberg E., Magnezi R. (2021). WhatsApp Tele-Medicine—Usage patterns and physicians views on the platform. Isr. J. Health Policy Res..

[26] Chang J.E., Lai A.Y., Gupta A., Nguyen A.M., Berry C.A., Shelley D.R. (2021). Rapid transition to telehealth and the digital divide: Implications for primary care access and equity in a post-COVID era. Milbank Q..

[27] Zanaboni P., Fagerlund A.J. (2020). Patients’ use and experiences with e-consultation and other digital health services with their general practitioner in Norway: Results from an online survey. BMJ Open.

[28] Pierce R.P., Stevermer J.J. (2020). Disparities in use of telehealth at the onset of the COVID-19 public health emergency. J. Telemed. Telecare.

[29] Kaufman-Shriqui V., Shani M., Boaz M., Lahad A., Vinker S., Birk R. (2022). Opportunities and challenges in delivering remote primary care during the Coronavirus outbreak. BMC Prim. Care.

[30] Singer A., Kosowan L., LaBine L., Shenoda D., Katz A., Abrams E.M., Halas G., Wong S.T., Talpade S., Kirby S. (2022). Characterizing the use of virtual care in primary care settings during the COVID-19 pandemic: A retrospective cohort study. BMC Prim. Care.

[31] Juergens N., Huang J., Gopalan A., Muelly E., Reed M. (2022). The association between video or telephone telemedicine visit type and orders in primary care. BMC Med. Inform. Decis. Mak..

[32] Al-Mutairi A.M., Alshabeeb M.A., Abohelaika S., Alomar F.A., Bidasee K.R. (2023). Impact of telemedicine on glycemic control in type 2 diabetes mellitus during the COVID-19 lockdown period. Front. Endocrinol..

[33] Ufholz K., Sheon A., Bhargava D., Rao G. (2022). Telemedicine Preparedness Among Older Adults with Chronic Illness: Survey of Primary Care Patients. JMIR Form. Res..

[34] Khairat S., Meng C., Xu Y., Edson B., Gianforcaro R. (2020). Interpreting COVID-19 and Virtual Care Trends: Cohort Study. JMIR Public Health Surveill..

[35] Stamenova V., Agarwal P., Kelley L., Fujioka J., Nguyen M., Phung M., Wong I., Onabajo N., Bhatia R.S., Bhattacharyya O. (2020). Uptake and patient and provider communication modality preferences of virtual visits in primary care: A retrospective cohort study in Canada. BMJ Open.

[36] Dalbosco-Salas M., Torres-Castro R., Rojas Leyton A., Morales Zapata F., Henríquez Salazar E., Espinoza Bastías G., Beltrán Díaz M.E., Tapia Allers K., Mornhinweg Fonseca D., Vilaró J. (2021). Effectiveness of a Primary Care Telerehabilitation Program for Post-COVID-19 Patients: A Feasibility Study. J. Clin. Med..

[37] Reed M., Huang J., Graetz I., Muelly E., Millman A., Lee C. (2021). Treatment and follow-up care associated with patient-scheduled primary care telemedicine and in-person visits in a large integrated health system. JAMA Netw. Open.

[38] Laddu D., Ma J., Kaar J., Ozemek C., Durant R.W., Campbell T., Welsh J., Turrise S. (2021). Health behavior change programs in primary care and community practices for cardiovascular disease prevention and risk factor management among midlife and older adults: A scientific statement from the American Heart Association. Circulation.

[39] Gulati M., Levy P.D., Mukherjee D., Amsterdam E., Bhatt D.L., Birtcher K.K., Blankstein R., Boyd J., Bullock-Palmer R.P., Conejo T. (2021). 2021 AHA/ACC/ASE/CHEST/SAEM/SCCT/SCMR Guideline for the Evaluation and Diagnosis of Chest Pain: A Report of the American College of Cardiology/American Heart Association Joint Committee on Clinical Practice Guidelines. J. Am. Coll. Cardiol..

[40] Kelsey M.D., Nelson A.J., Green J.B., Granger C.B., Peterson E.D., McGuire D.K., Pagidipati N.J. (2022). Guidelines for cardiovascular risk reduction in patients with type 2 diabetes: JACC Guideline Comparison. J. Am. Coll. Cardiol..

[41] Han X., Chen W., Gao Z., Lv X., Sun Y., Yang X., Shan H. (2021). Effectiveness of telemedicine for cardiovascular disease management: Systematic review and meta-analysis. Ann. Palliat. Med..

[42] Jiang W., Majumder S., Kumar S., Subramaniam S., Li X., Khedri R., Mondal T., Abolghasemian M., Satia I., Deen M.J. (2022). A wearable tele-health system towards monitoring COVID-19 and chronic diseases. IEEE Rev. Biomed. Eng..

[43] Fisher K., Davey A.R., Magin P. (2022). Telehealth for Australian general practice: The present and the future. Aust. J. Gen. Pract..

[44] Carrillo de Albornoz S., Sia K.L., Harris A. (2022). The effectiveness of teleconsultations in primary care: Systematic review. Fam. Pract..

[45] Hammersley V., Donaghy E., Parker R., McNeilly H., Atherton H., Bikker A., Campbell J., McKinstry B. (2019). Comparing the content and quality of video, telephone, and face-to-face consultations: A non-randomised, quasi-experimental, exploratory study in UK primary care. Br. J. Gen. Pract..

[46] Morris C., Scott R.E., Mars M. (2021). WhatsApp in clinical practice-the challenges of record keeping and storage. A scoping review. Int. J. Environ. Res. Public Health.

[47] Pallarés Carratalá V., Górriz-Zambrano C., Llisterri Caro J.L., Gorriz J.L. (2020). The COVID-19 pandemic: An opportunity to change the way we care for our patients. Semergen.

[48] Taylor P., Berg C., Thompson J., Dean K., Yuan T., Nallamshetty S., Tong I. (2022). Effective access to care in a crisis period: Hypertension control during the COVID-19 pandemic by telemedicine. Mayo Clin. Proc. Innov. Qual. Outcomes.

[49] Ye S., Anstey D.E., Grauer A., Metser G., Moise N., Schwartz J., Kronish I., Abdalla M. (2022). The impact of telemedicine visits on the controlling high blood pressure quality measure during the COVID-19 pandemic: Retrospective cohort study. JMIR Form. Res..

[50] Kassavou A., Wang M., Mirzaei V., Shpendi S., Hasan R. (2022). The association between smartphone app-based self-monitoring of hypertension-related behaviors and reductions in high blood pressure: Systematic review and meta-analysis. JMIR mHealth uHealth.

[51] Arshad Ali S., Bin Arif T., Maab H., Baloch M., Manazir S., Jawed F., Kumar Ochani R. (2020). Global Interest in Telehealth During COVID-19 Pandemic: An Analysis of Google Trends™. Cureus.

[52] (2020). Coronavirus Disease (COVID-19) Weekly Epidemiological Updates and Monthly Operational Updates. https://www.who.int/emergencies/diseases/novel-coronavirus-2019/situation-reports.

[53] Saigí-Rubió F., Nascimento I.J.B.D., Robles N., Ivanovska K., Katz C., Azzopardi-Muscat N., Ortiz D.N. (2022). The current status of telemedicine technology use across the World Health Organization European Region: An overview of systematic reviews. J. Med. Internet Res..

[54] Peterson S., Young J., King V., Meadows J. (2022). Patient expectations for synchronous telerehabilitation visits: A survey study of telerehabilitation-naive patients. Telemed. J. e-Health.

[55] Fakhri G., Assaad S., Chaaya M. (2020). Hypertension prevalence and control among community-dwelling lebanese older adults. J. Clin. Hypertens..

[56] Muli S., Meisinger C., Heier M., Thorand B., Peters A., Amann U. (2020). Prevalence, awareness, treatment, and control of hypertension in older people: Results from the population-based KORA-age 1 study. BMC Public Health.

[57] Rabi D.M., McBrien K.A., Sapir-Pichhadze R., Nakhla M., Ahmed S.B., Dumanski S.M., Butalia S., Leung A.A., Harris K.C., Cloutier L. (2020). Hypertension Canada’s 2020 Comprehensive Guidelines for the Prevention, Diagnosis, Risk Assessment, and Treatment of Hypertension in Adults and Children. Can. J. Cardiol..

[58] Whelton P.K., Carey R.M., Aronow W.S., Casey D.E., Collins K.J., Dennison Himmelfarb C., DePalma S.M., Gidding S., Jamerson K.A., Jones D.W. (2018). 2017 ACC/AHA/AAPA/ABC/ACPM/AGS/APhA/ASH/ASPC/NMA/PCNA Guideline for the Prevention, Detection, Evaluation, and Management of High Blood Pressure in Adults: A Report of the American College of Cardiology/American Heart Association Task Force on Clinical Practice Guidelines. Hypertension.

[59] Lahat A., Shatz Z. (2021). Telemedicine in clinical gastroenterology practice: What do patients prefer?. Ther. Adv. Gastroenterol..

