# Supplementary material for: Telemedicine in Elderly Hypertensive and Patients with Chronic Diseases during the COVID-19 Pandemic: A Systematic Review and Meta-Analysis
Source: J Clin Med. 2023 Sep 24;12(19):6160. doi: 10.3390/jcm12196160 (PMC10574013; doi:10.3390/jcm12196160)
Supplement: Supplementary file 1 [file jcm-12-06160-s001.zip › jcm-2609595-supplementary.pdf]

Suplementary Table S1. Critical reading of included studies.

Study	Question 1	Question 2	Question 3
Gomes-de Almeida et al., 2021 (Portugal) [22]	Y	Y	Y
Eberly et al., 2020 (USA) [21]	Y	Y	Y
Summers et al., 2022 (UK) [20]	Y	Y	Y
Kaufman-Shriqui et al., 2022 (Israel) [29]	Y	Y	Y
Lee et al., 2022 (Korea) [19]	Y	Y	Y
Rodríguez-Fortúnez et al., 2019 (Spain) [23]	Y	Y	Y
Pierce and Stevermer 2020 (USA) [28]	Y	Y	Y
Barayev et al., 2021 (Israel) [25]	Y	Y	Y
Chang et al., 2021 (USA) [26]	Y	Y	Y
Zanaboni and Fagerlund, 2020 (Norway) [27]	Y	Y	Y
Dopelt et al., 2021 (Israel) [24]	Y	Y	Y
Singer A. et al., 2022 (Canada) [30]	Y	Y	Y
Juergens N. et al., 2022 (USA) [31]	Y	Y	Y
Al-Mutairi A. M. et al., 2023 (Saudi Arabia) [32]	Y	Y	Y
Ufholz K. et al., 2023 (USA) [33]	Y	Y	Y
Khairat S. et al., 2020 (USA) [34]	Y	Y	Y
Stamenova V. et al., 2020 (Canada) [35]	Y	Y	Y
Dalbosco-Salas M. et al., 2021(Chile) [36]	Y	Y	Y

Note: Question 1= Did the study address a clearly focused issue? ; Question 2= Was the sample recruited in an acceptable way?; Question 3= Waere the outcomes accurately measured to minimise bias? ; Y= yes; N= No.
